# COVID19 infection in a patient with paroxysmal nocturnal hemoglobinuria: A case report

**DOI:** 10.1097/MD.0000000000025456

**Published:** 2021-05-21

**Authors:** Juraj Sokol, Frantisek Nehaj, Michal Mokan, Lenka Lisa, Jan Stasko

**Affiliations:** a Department of Hematology and Transfusion Medicine, National Centre of Hemostasis and Thrombosis; b First Department of Internal Medicine, Jessenius Faculty of Medicine in Martin, Comenius University in Bratislava, Martin, Slovakia.

**Keywords:** case report, complement, COVID19, paroxysmal nocturnal hemoglobinuria, treatment, thrombosis

## Abstract

**Introduction:**

Paroxysmal nocturnal hemoglobinuria (PNH) is an acquired, life-threatening hemopoietic stem cell disorder characterized by the triad of hemolytic anemia, thrombosis, and impaired bone marrow function. Evidence suggests that severe outcomes in COVID19 infection are attributed to the excessive activation of the complement cascade leading to acute lung injury and associated is with an increased prothrombotic state.

**Patient concerns:**

A 27-year-old Caucasian man with PNH presented to the Emergency Department of our hospital with acute onset shortness of breath, cough and blood in urine.

**Diagnosis:**

The patient was diagnosed with acute hemolytic exacerbation of PNH complicated with moderate COVID19 pneumonia.

**Outcomes:**

The patient was initiated with an anticoagulant unfractionated heparin, dexamethasone, and cefuroxime injection. His symptoms quickly resolved, and he was discharged after 5 days.

**Conclusion:**

The complement system activation is a critical component in the sequalae of COVID19 infection. Evidence suggests that severe outcomes in COVID19 infection are attributed to the excessive activation of the complement cascade leading to acute lung injury and associated is with an increased prothrombotic state. Notably, C5a concentration was noted to be higher in patients with COVID19 infection. The use of complement inhibitors to attenuate immune mediated damage in COVID19 nevertheless represents a very interesting theoretical approach. However, careful consideration as to which patients may benefit will be required and the outcome of clinical trials needed.

## 1 Introduction

Paroxysmal nocturnal hemoglobinuria (PNH) is an acquired, life-threatening hemopoietic stem cell disorder characterized by the triad of hemolytic anemia, thrombosis, and impaired bone marrow function. PNH arises due to an acquired mutation in the synthesis of glycosylphosphatidylinositol (GPI) anchor protein, which leads to a deficiency of complement regulatory proteins and unregulated complement-mediated hemolysis.^[1]^ The world incidence of PNH is still unknown, but it is estimated to be 1 to 5 cases per million inhabitants in the USA, a much lower incidence than that of bone marrow aplasia, whose prevalence is 5 to 10 times higher. Still about statistics in the USA, prevalence does not vary by sex or race/ethnicity. The same occurs in some Asian countries, such as Thailand, Japan, and the Far East, where PNH has lower incidence than bone marrow aplasia. As regards sex, in Europe, PNH is more common in women, whereas in Asia it is more common in men. PNH can occur at any age, but it is generally diagnosed between third and fifth decades.^[2–5]^ Patients experience intravascular hemolysis, smooth muscle dystonia, renal failure, arterial and pulmonary hypertension, recurrent infectious diseases, and an increased risk of notably dreadful thrombotic complications.^[6]^

The diagnosis of PNH is made by means of clinical findings and laboratory tests to confirm the degree of hemolysis (haptoglobin, lactate dehydrogenase, direct Coombs test, reticulocyte count and total bilirubin and bilirubin fraction) and deficiency of anchored proteins of the complement system (CD55, CD59, and FLAER) in granulocytes (CD15, CD33, and CD24) and monocytes (CD14 and CD64) by flow cytometry, the gold standard method.^[6–8]^ Management of PNH has been dramatically revolutionized by the development of eculizumab, which brings benefits in terms of hemolysis, quality of life, renal function, thrombotic risk, and life expectancy. Eculizumab is a humanized monoclonal antibody. It acts as a complement inhibitor, binding specifically to complement protein C5 with high affinity, thereby inhibiting cleavage to C5a, a prothrombotic and proinflammatory molecule and C5b, preventing the generation of the terminal complement complex C5b-9. Prophylaxis and treatment of arterial and venous thrombosis currently remain a challenge in PNH.^[6]^ The objective of this study was to report the rare case of a PNH patient with COVID-19 pneumonia, strategies to diagnose, and therapeutic challenges.

## 2 Case report

A 27-year-old Caucasian man presented to the Emergency Department of our hospital with acute onset shortness of breath, cough, and blood in urine. Rapid antigen tests were positive for SARS-CoV-2 infection. He did not have any fever, chills, wheezing, sputum production, chest pain, palpitations, pressure, abdominal pain, abdominal distension, nausea, vomiting, and diarrhea.

Breathing problems had begun approximately 2 days before admission and had progressively worsened with no associated, aggravating, or relieving factors noted. He also complained of a paroxysmal productive cough of indeterminate origin that he had noticed 7 days earlier. On the day of admission, he had noticed blood in urine.

The past medical history was significant in that at the age of 26 years he presented to the Emergency Department with dark urine. He was otherwise previously healthy. Since the urine problem resolved spontaneously and the patient was under the influence of alcohol at the time of this event, no further differential diagnosis was made. However, the situation repeated in 2 months. During this hospitalization, the diagnosis of PNH was made. The PNH clone was positive with erythrocytes 0.7%, 8.89% for monocytes, and 13.25% for granulocytes. Bone marrow smears were normocellular and there were only mild signs of dysmegacaryocytopoiesis without excess blasts (2%). Karyotype was normal. After the 6-month follow-up period, hemolytic anemia still persisted, but there was no need for transfusions. The patient was scheduled to start treatment with eculizumab. At the time of diagnosis of COVID19, the patient was waiting for the medical insurance approval of eculizumab treatment. Past surgical history is only significant for a right knee arthroscopy.

The patient's weight was 80 kg, and his body mass index was 24.0 kg/m^2^. His skin, skin adnexa, and mucosa showed no pathological changes. Neurological, cardiac, abdominal, and locomotor system examinations were normal with mild crackles in the right lung base. Blood pressure on admission was 120/70 mm Hg, heart rate was 92 beats/min, and the patient had a regular body temperature of 36.8 °C. The patient's respiratory rate was 12 breaths/min and SPO_2_ was 92% on room oxygen.

The blood test results showed hemoglobin of 9.8 g/dL (normal range adult male, 14–17.9 g/dL), white blood cell count of 12.3 × 10^9^/L (normal range, 3.9–10 × 10^9^/L), platelets of 118 × 10^9^/L (normal range, 140–400 × 10^9^/L), lymphocytes of 1.3 × 10^9^/L (normal range, 1.2–3.4 × 10^9^/L), reticulocytes 299 × 10^9^/L (normal range, 40–105 × 10^9^/L), total bilirubin 93 μmol/L (normal range, 5–21 μmol/L), haptoglobin < 0.08 g/L (normal range, 0.35–2.5 g/L), and lactate dehydrogenase 44 μkat/L (normal range, 1.83–4.12 μkat/L). Other investigations such as prothrombin time, activated partial thromboplastin time, international normalized ratio, D-dimer and liver enzymes were normal (see Table 1). C-reactive protein was slightly elevated 18.5 mg/L (normal range,0.0–5.0 mg/L). PNH clone did not change. COVID19 test using targeted rich multiplex (RT) polymerase chain reaction of nasopharyngeal swab came back positive for SARS-CoV-2 infection.

**Table 1:** Changes of selected parameters (blood count and biochemical parameters) over time.

Selected parameters	Value at the time of admission	Value at the time of discharge	Two wk after discharge	Local references
Leukocytes (×10^9^/L)	12.3	5.6	7.5	3.9–10
Neutrophils (×10^9^/L)	10.5	3.5	4.5	1.4–6.5
Lymphocytes (×10^9^/L)	1.3	1.3	2	1.2–3.4
Hemoglobin (g/dL)	9.8	9.9	10.5	14.0–17.9
Platelets (×10^9^/L)	118	142	189	140–400
Reticulocytes (×10^9^/L)	299	254	200	40–105
Creatinine (μmol/L)	98	83	70	59–104
Total bilirubin (μmol/L)	93	42	38	5–21
Conjugated bilirubin (μmol/L)	10.2	7.5	6	0.1–3.4
Haptoglobin (g/L)	< 0.08	< 0.08	2.2	0.35–2.5
C-reactive protein (mg/L)	18.5	6.9	1.0	0.0–5.0
Lactatdehydrogenase (μkat/L)	44	29	15	1.83–4.12
D-dimer (mg/L)	0.4	0.4	0.4	<0.5

**Figure 1:**
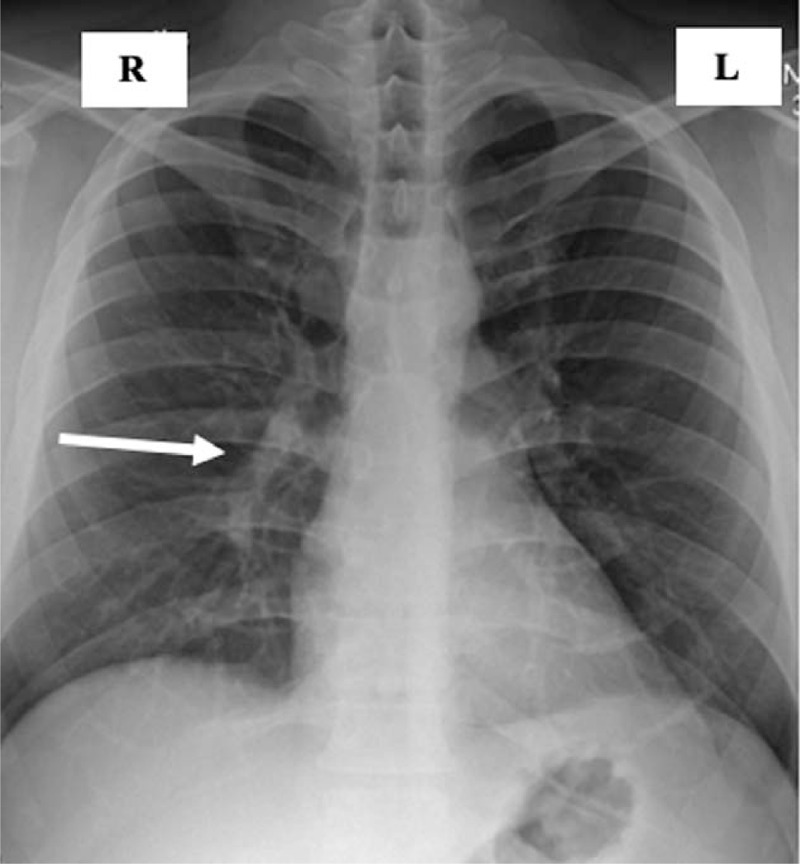
Chest X-ray on hospital admission. Inflammatory changes are observed in the middle and lower lung fields on the right (white arrow).

A chest radiograph displayed bronchopneumonia at the right lower lobe in the lower and middle segments (Fig. 1), and the ECG showed sinus tachycardia.

The patient was diagnosed with acute hemolytic exacerbation of PNH complicated with moderate COVID19 pneumonia.

The patient was initiated with an anticoagulant prophylactic subcutaneous Low Molecular Weight Heparin (enoxaparin 4000 UI anti-Xa, daily). Dexamethasone (6 mg intravenously every 24 hours) and cefuroxime injection (1.5 g intravenously every 8 hours) were added to the treatment.

**Figure 2:**
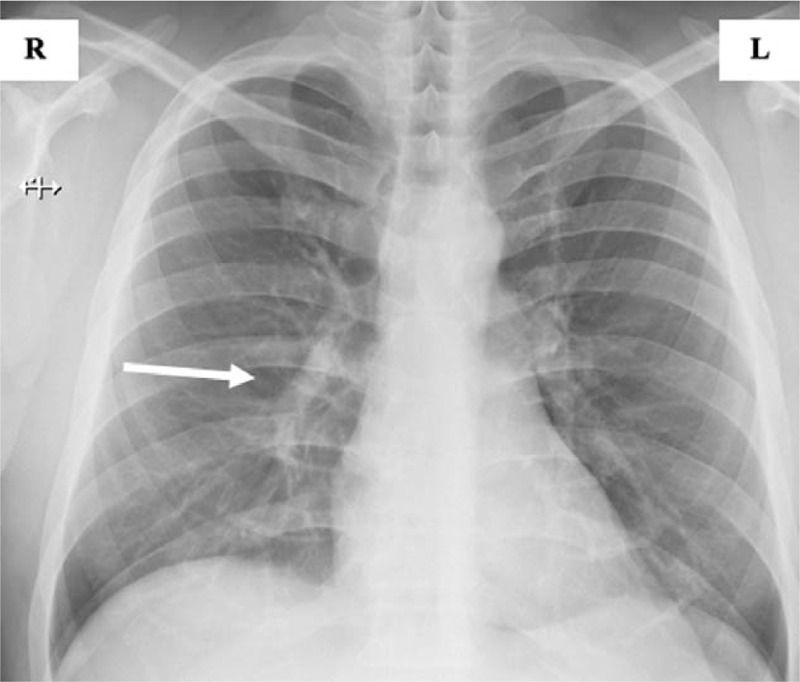
Chest X-ray before hospital discharge. Regression of inflammatory changes in the middle and lower lung field on the right (white arrow).

His symptoms quickly resolved, and he was discharged after 5 days. Hemolytic activity persists as before SARS-CoV-2 infection (see Table 1 & Fig. 2). Of note, COVID-19 RT-polymerase chain reaction testing remained positive for the next 3 months. Although the patient was discharged without anticoagulant or antiplatelet therapy, he had not yet experienced with a thrombotic event.

## 3 Discussion

PNH is a clonal disorder of hematopoietic progenitor cells caused by an acquired mutation of the X-linked phosphatidylinositol glycans class A gene.^[9]^ The absence of glycosylphosphatidylinositol anchored complement regulatory proteins CD55 and CD59 from the membrane of circulatory cells is responsible for activation of the complement system on the surface of the red cell membrane. This leads to complement mediated intravascular hemolysis, activation of platelets, and the coagulation cascade resulting in a hypercoagulable state.^[9]^ PNH, although rare, can be fatal and includes an increased risk of thromboembolism and severe end-organ damage. Approximately, 35% of patients die within 5 years if untreated due to thrombosis and related complications.^[10,11]^

The complement system activation is a critical component in the sequalae of COVID19 infection. Evidence suggests that severe outcomes in COVID19 infection are attributed to the excessive activation of the complement cascade leading to acute lung injury and associated is with an increased prothrombotic state.^[12–14]^ Notably, C5a concentration was noted to be higher in patients with COVID19 infection.^[12,15]^ Complement blockers can be used as potential therapeutic targets in COVID19 patients.^[16,17]^ In addition, there is no evidence of increased susceptibility to SARS-CoV-2 in patients on anticomplement therapy.^[18]^ On the other hand, there is also evidence that eculizumab administered in a classical schedule for PNH did not contribute to control a severe course of COVID-19.^[19]^

The use of complement inhibitors to attenuate immune mediated damage in COVID-19 nevertheless represents a very interesting theoretical approach. However, careful consideration as to which patients may benefit will be required and the outcome of clinical trials needed.

It is also important to note, that both diseases are known to cause hypercoagulability-related morbidity and mortality separately. Further research should also focus on anticoagulant therapy. Due to the mild course of COVID19, our patient received a prophylactic dose of Low Molecular Weight Heparin.

## 4 Conclusion

Here we report 1 case of COVID19 infection requiring hospitalization. As mentioned above, there is evidence of increased activation of coagulation pathways and susceptibility to thromboembolic disease in patients with COVID19, though we did not detect any thrombotic complications in this case. Infections increase risk of breakthrough hemolysis in PNH due to increased activation of the complement system. COVID19 induced breakthrough hemolysis was seen in our case report.

## Author contributions

**Conceptualization:** Juraj Sokol, Frantisek Nehaj.

**Data curation:** Frantisek Nehaj.

**Formal analysis:** Frantisek Nehaj.

**Funding acquisition:** Juraj Sokol, Michal Mokan.

**Investigation:** Michal Mokan.

**Methodology:** Michal Mokan.

**Project administration:** Michal Mokan.

**Resources:** Lenka Lisa.

**Software:** Michal Mokan, Lenka Lisa.

**Supervision:** Juraj Sokol, Lenka Lisa.

**Validation:** Lenka Lisa.

**Visualization:** Juraj Sokol, Michal Mokan, Jan Stasko.

**Writing – original draft:** Juraj Sokol, Frantisek Nehaj, Michal Mokan.

**Writing – review & editing:** Lenka Lisa, Jan Stasko.
